# Slow acoustic surface modes through the use of hidden geometry

**DOI:** 10.1038/s41598-021-01269-4

**Published:** 2021-11-10

**Authors:** S. R. Shelley, J. G. Beadle, A. P. Hibbins, J. R. Sambles

**Affiliations:** Electromagnetic and Acoustic Materials Group, University of Exeter, Exeter, EX4 4QL UK

**Keywords:** Physics, Acoustics

## Abstract

The acoustic surface modes supported by a partly covered periodic meander groove structure formed in an assumed perfectly rigid plate are investigated. This allows one to create a slower acoustic surface wave than can be achieved with the same uncovered meander structure. By changing the size of the uncovered section the phase and group speeds can be tuned. When the uncovered section of the meander structure is centred along the grooves then the distance along the grooves between neighbouring holes is the same on both sides of the structure so no band gap is observed at the first Brillouin zone boundary due to glide symmetry. This then gives quite linear dispersion. As the uncovered section’s position is moved away from the centre of the meander structure a band gap opens at the Brillouin zone boundary.

## Introduction

In recent years controlling sound propagation over surfaces through the use of patterning has attracted substantial interest^1,2^ in part dealing with acoustic surface waves (ASWs)^3–5^ which can be utilised for applications such as sound collimation^6^ or attenuation^7^. Sub-wavelength structuring has also been shown to be able to influence the propagation of sound over the surface^8,9^. One type of subwavelength metamaterial is labyrinthine arrays^10–12^ which have been shown to create high effective refractive indices (slow phase speed) by coiling up space. By controlling the path length of these structures one is able to tune the effective acoustic refractive index. This can then be used for example to create acoustic lenses^13^ or even materials with a negative index^11^. One issue with these structures is that applications generally require a broadband frequency response but this can be difficult to achieve due to mode dispersion as one approaches the Brillouin zone boundary of these periodic structures. A solution to this is to create a glide symmetric structure^14,15^. Such surfaces have reflection symmetry when the unit cell is shifted by half the periodicity. These have a degeneracy of states at the Brillouin zone boundary so can have a near linear dispersion across the boundary^16^. Recent work has examined the ASWs supported by an uncovered meander structure^17^ finding slow ASWs. Due to the glide symmetry of the surface these exhibit broadband near constant group speed. In the work presented here, the ASWs supported by a partly covered meander structure are investigated. These demonstrate lower group and phase speeds than the uncovered case allowing one to have slower ASWs without increasing the length of the meander structure.

## Experimental

### Sample design

**Figure 1:**
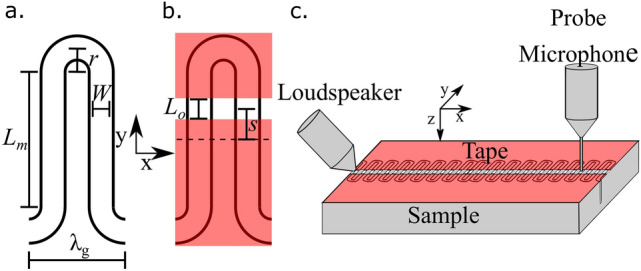
(**a**) Schematic of the unit cell of the uncovered meander structure used. This consists of a single continuous air filled channel surrounded by an assumed perfectly rigid material. (**b**) Schematic of the covered meander structure. The underlying structure is the same except the unit cell has been partially covered (red regions) leaving a small uncovered region with centre shifted a distance *s* from the centre of the structure. Both structures extend into the page a depth *d*. (**c**) Experimental setup. A loud speaker emits a pulse of sound that excites an acoustic surface wave. A probe microphone scans over the surface detecting the signal.

A schematic of the unit cell of the meander structures is shown in Fig. 1a. This consists of two straight channels of length $L_m$, width *W*, and depth *d* that are connected by semicircular channel sections of radius *r* on alternate ends forming a unit cell of size $\lambda _{{\text{{g}}}}$ in the $\hat{\text{{x}}}$ direction. These channels are air-filled and created by milling out a solid aluminium block that measures 600 mm long in x, 110 mm wide in y and 8 mm deep in z. The properties and dispersion of the modes supported by such a structure are discussed at length in the work of Beadle et al.^17^. In this present study the meander structure is partially covered by a thin layer of acoustically rigid parcel tape, leaving a straight strip of length $L_o$ open, located a distance *s* from the centre of the structure. This creates a periodic array of openings (holes) to the meander structure, which is illustrated in Fig. 1b. Note that this results in two openings per meander-structure unit cell. On the air side of the structure the array of openings has a spacing of $\lambda _g/2$, however on the substrate side the repeat distance covers two openings. The meander path length between unit cells, $\lambda _c$, is given by,

1 $$\begin{aligned} \lambda _{c} = 2L_m + 2\pi r. \end{aligned}$$

**Figure 2:**
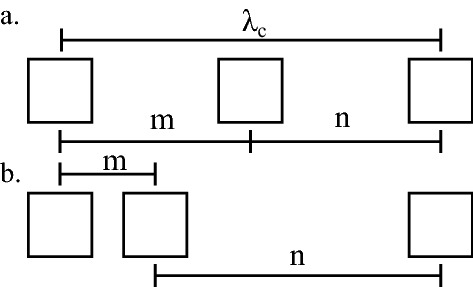
Apparent spacing of cavities in the partly covered meander structure. Here $\lambda _{c}$ is the path length along the meander between neighbouring unit cells and *m* and *n* is the distance between neighbouring cavities with $m + n = \lambda _{c}$. (**a**) The uncovered section is aligned with the centre of the meander structure ($s = 0$). In this case $m = n$. (**b**) The uncovered section unaligned with the centre of the meander structure ($s \ne 0$).

A schematic of the spacing between the openings based on the meander structure are shown in Fig. 2. This spacing is subdivided into two lengths denoted as *m* and *n* which characterise the distance between neighbouring holes with $m + n = \lambda _{c}$. When the uncovered section is aligned with the centre of the meander structure ($s = 0$) a special case is realised with $m = n$. The full geometry of the problem is shown in Fig. 1c. Here the meander structure is repeated in the ${\hat{x}}$ direction and extends in the ${\hat{y}}$ direction. Three base samples with different size meander structures were utilised in the experiments. Two of the samples have $\lambda _g = 4$ mm, $W = 1$ mm with (1) $L_m = 10$ mm and (2) $L_m= 5$ mm. The third sample (3) has $\lambda _g = 6$ mm, $W = 2$ mm with $L_m = 5$ mm. These samples are covered with acoustically rigid tape so that a central hole of 1 mm is left exposed along the structure. For sample (2) the position of the hole in y along the straight section is also varied. The dispersion of the surface waves supported by these structures are measured using the technique discussed in the “Methods” section.

### Results

**Figure 3:**
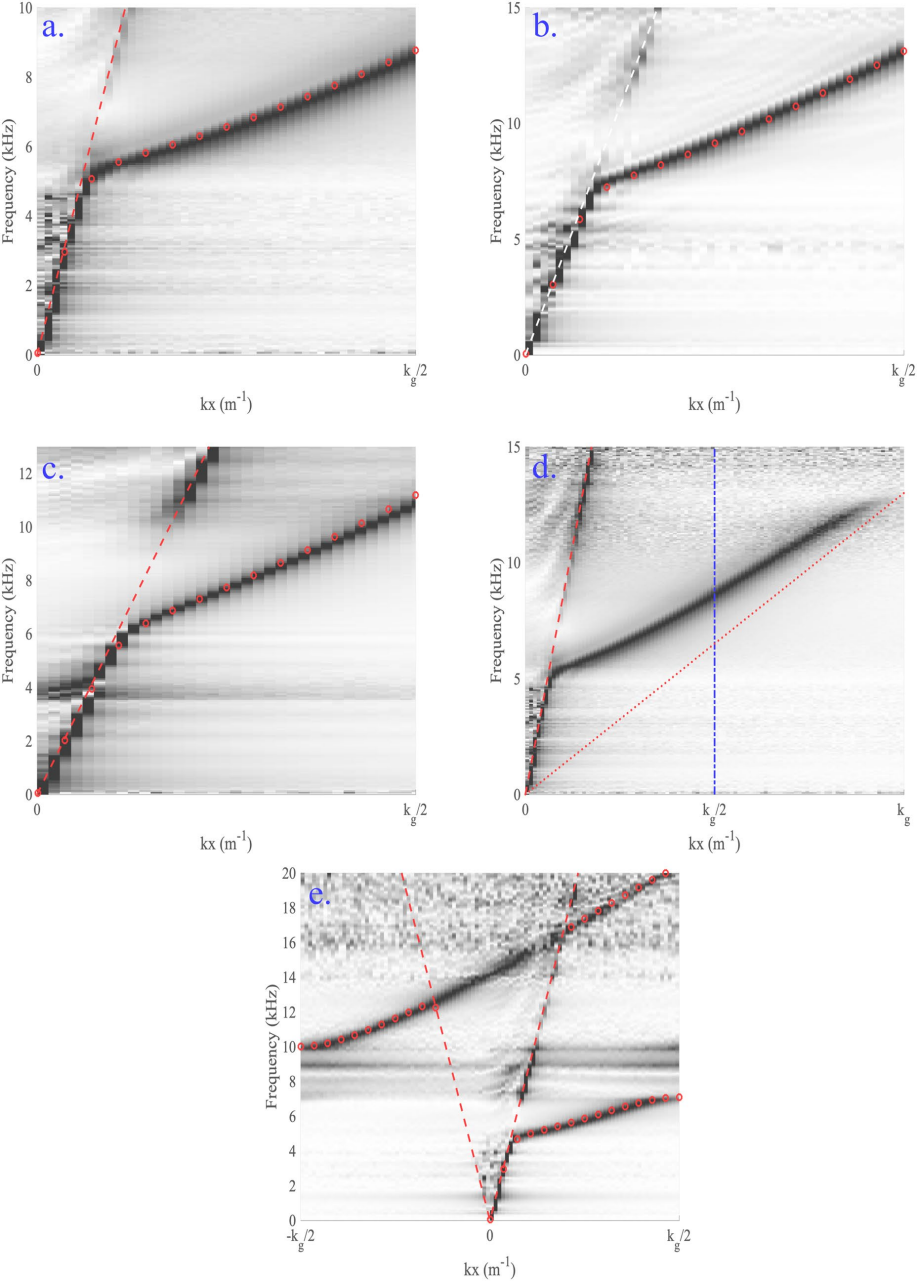
Dispersions of the partly covered meander structures. Grey scale shows experimental data, and red circles the modelled results. The red dashed line shows the free-space sound-lines. (**a**–**c**). The experimental dispersions for the three samples in the first Brillouin zone, (**a**) $\lambda _g = 4$ mm, $W = 1$ mm, $L_m = 10$ mm, $L_o = 0.8$ mm and $s = 0$ mm, (**b**) $\lambda _g = 4$ mm, $W = 1$ mm, $L_m = 5$ mm, $L_o = 0.8$ mm and $s = 0$ mm, (**c**) $\lambda _g = 6$ mm, $W = 2$ mm with $L_m = 5$ mm, $L_o = 0.8$ mm and $s = 0$ mm. (**d**) The dispersion presented in (**a**) extended past the first Brillouin zone boundary (dot dashed blue line) also showing the meander sound-line (red dotted line). (**e**) Data for the sample used in (**a**) with $L_o = 0.5$ mm, $s = 3.6$ mm.

**Figure 4:**
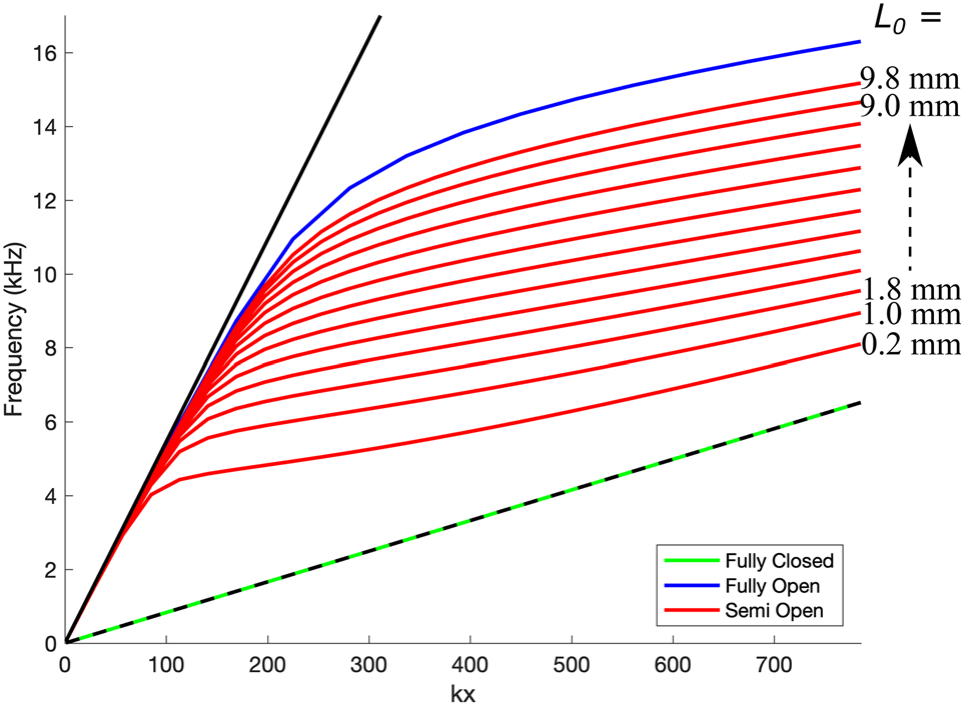
Comparison between dispersions of the partly open meander (solid red) as the uncovered length $L_o$ is varied between 0.2 mm and 9.8 mm in steps of 0.8 mm. The meander structure has $L_m = 10$ mm, $W = 2$ mm, $\lambda _g = 4$ mm and $d = 5$ mm. The solid and dashed black lines show the free space and meander sound lines respectively. The fully closed (green solid) and fully open (solid blue) cases are also shown.

Figure 3a–c shows the experimental dispersions for each of the partly covered meander structures with the uncovered section aligning with the centre of the meander structure. Excellent agreement can be seen between the modelled results (modelled using COMSOL) and the experimental data. Due to the nature of the experimental scan only signals in the positive $k_{x}$ direction are detected. In each of these dispersions a mode is found close to the free-space sound-line at small $k_{x}$. This shows that at low frequency the sound is barely interacting with the meander structure and is just a grazing sound wave in free space. As $k_{x}$ increases the mode begins to disperse away from the free space sound line. The mode’s group velocity starts to decrease towards the effective speed of sound in the meander. This effective speed of sound is reduced compared to the free space speed due to the increased path length the sound in the meander structure has to travel compared to the distance in free space. At the Brillouin zone boundary no band gap is observed in Fig. 3a–c for any of the samples shown. This is because, as discussed previously, there are two holes per unit cell and the distance between neighbouring cavities is constant, creating a glide symmetric surface. The result is a degeneracy of the modes at the Brillouin zone boundary and thus a band gap can not exist. Figure 3d shows the dispersion for the sample with $\lambda _g = 4$ mm, $W = 1$ mm $L_m = 10$ mm and $L_o = 0.8$ mm extended past the first Brillouin zone. Here the sound line based on the effective speed of sound in the meander is also shown. From this dispersion one can see that the mode continues through the Brillouin zone boundary as would be expected. It can also clearly be seen that the mode does tend towards the meander sound line. This shows that at large $k_x$ values the mode is more confined within the meander structure and will have approximately a constant phase and group speed. To understand the behaviour of the partly covered meander structure it is first useful to consider the two extreme cases, fully covered and fully uncovered. The case of the fully uncovered meander was examined by Beadle et al.^17^ and it was shown that the meander structure acted like a waveguide to first order with a dispersion given by

2 $$\begin{aligned} f = \frac{v}{2\pi }\sqrt{\left( \frac{k_{x}}{n_{\text{wg}}}\right) ^2 + k_{z}^2 }, \end{aligned}$$

where *f* is the frequency and *v* is the speed of sound in air. Here $n_{wg}$, the effective index of the waveguide, is given by $\lambda /\lambda _g$ and $k_{z}$ is given by $2\pi /4d$ which is the quarter wavelength resonance associated with the depth of the meander (ignoring end effects). The interplay of the meander path length term and the depth resonance term in this equation leads to dispersions such as the blue line in Fig. 4. At small $k_{x}$ the dispersion is dominated by the depth of the meander and thus follows the soundline. As the value of $k_{x}$ increases the mode may interact more with the meander so transitions from the free space sound line to the waveguide mode of the meander given by Eq. (2). However in the fully closed case the lowest order mode of effective waveguide created by the meander is no longer a quarter wavelength in z but starts at zero frequency. The resulting dispersion diagram just shows a mode travelling along the effective sound line created by the increased path length of the meander compared to the unit cell size, the green line in Fig. 4. In the partially covered case presented here a small portion of the meander is open to free space whilst the rest is covered. Beneath the openings the effective waveguide supports a quarter wavelength resonance whilst under the covered sections the lowest mode supported is just sound travelling further by being in the guide but with no lower cut-off. Thus the overall effective wave guide mode is a hybrid of the two cases, lying somewhere between them depending on the size of the holes compared to the overall meander structure. Similar to the fully open case, the dispersion observed for the semi open case transitions from the free space soundline to the effective waveguide mode as more of the sound is able to interact with the meander structure. As the dispersion away from the free space sound line is determined by the ratio of open to closed area of the meander one is able to tune the dispersion between the two extreme cases by varying the size of the hole in the meander covering. Figure 4 shows modelled dispersions of the meander structure as $L_o$, the size of the hole, is varied. This is based on the meander structure with $L_m = 10$ mm $W = 2$ mm $\lambda _g = 4$ mm $d = 5$ mm with $L_o$ varied between 0.2 and 9.8 mm. One can see that as the hole size is increased then the dispersion tends towards that of the fully open case. Similarly for the smallest hole sizes the dispersion tends towards the fully closed case. Thus by varying the hole size both the phase and group speeds of the mode can be controlled.

As discussed previously by changing the location of the uncovered section relative to the meander structure one is able to break the glide symmetry of the system. Thus by moving the position of the uncovered section one can open a band gap at the Brillouin zone boundary. This is similar to a 1D Peierls distortion^18^, although in this case the spacing between the uncovered sections is only distorted on the substrate side, whereas on the air side the openings remain equidistant. The size of this band gap is dependent on how far from the centre this section is shifted. Note that if it is shifted to align with either end of the meander then the system reduces to one hole per unit cell so the band gap closes again. Figure 3e shows the dispersion for sample (1) with the uncovered section shifted by 3.6 mm away from the centre of the meander. Again there is excellent agreement between the experimental data and the modelling results. Here the band gap at the first Brillouin zone boundary can clearly be seen.

## Conclusions

In this study the acoustic surface waves supported by partly-covered (hidden) meander structures have been investigated. A simple rigid tape covering is used to provide an easily controlled route to manipulating the phase and group velocities of the supported ASW. This allows one to markedly reduce the group and phase speed of the supported mode compared to the fully open case. By varying the size of the uncovered section one is able to tune the mode between the two extremes of fully open to fully covered. When the uncovered section is aligned with the centre of the meander structure no band gap is observed at the Brillouin zone boundary due to the glide symmetry of the structure. This leads to a wide frequency band with near constant group velocity. However as the uncovered section is shifted the glide symmetry of the system is broken, resulting in the creation of a band gap. The size of the band gap can be tuned by varying how much the uncovered section is shifted. This has potential applications if one desires a surface mode with both low group and phase speeds.

## Methods

To experimentally measure the dispersion, the technique described by Beadle et al.^17^ was used. A schematic of the experimental setup is shown in Fig. 1c. Here a loudspeaker with a conical attachment was positioned close to one end of the sample such that the produced sound was diffracted along the surface. In this work a Gaussian envelope sound pulse centred around 15 kHz was utilised. The signal was detected by a needle microphone located 0.2 mm above the surface that was scanned along the length of the sample in 0.5 mm increments over a total scan length of 400 mm. An average of three pulses was taken at each location to improve the signal to noise ratio. For each measured location a Fourier transform in time was performed on the detected signal to obtain the amplitude and phase for each frequency. A Fast Fourier Transform is space was then performed for each frequency to produce a dispersion diagram of the supported ASWs.

Simulations were also performed using a finite element method model in COMSOL Multiphysics^19^ using the pressure acoustics module.
